# Testing non-inferiority of blended versus face-to-face cognitive behavioural therapy for severe fatigue in patients with multiple sclerosis and the effectiveness of blended booster sessions aimed at improving long-term outcome following both therapies: study protocol for two observer-blinded randomized clinical trials

**DOI:** 10.1186/s13063-019-3825-2

**Published:** 2020-01-20

**Authors:** Marieke Houniet-de Gier, Heleen Beckerman, Kimberley van Vliet, Hans Knoop, Vincent de Groot

**Affiliations:** 1 Department of Rehabilitation Medicine, MS Center Amsterdam, Amsterdam Neuroscience Research Institute, Amsterdam University Medical Centers, Vrije Universiteit, P.O. Box 7057, 1007 MB Amsterdam, The Netherlands; 2 Department of Medical Psychology, MS Center Amsterdam, Amsterdam Neuroscience Research Institute, Amsterdam University Medical Centers, Vrije Universiteit, P.O. Box 7057, 1007 MB Amsterdam, The Netherlands; 3 Department of Medical Psychology, Amsterdam Public Health Research Institute, Amsterdam University Medical Centers, University of Amsterdam, Amsterdam, The Netherlands

**Keywords:** Multiple sclerosis, Fatigue, Cognitive behavioural therapy, Long-term effectiveness, Booster sessions, Blended care, Internet, Study protocol, Randomised clinical trial, Non-inferiority trial

## Abstract

**Background:**

Cognitive behavioural therapy (CBT) has been found to be effective in reducing fatigue severity in MS patients directly following treatment. However, long-term effects are inconsistent leaving room for improvement. In addition, individual face-to-face CBT draws heavily on limited treatment capacity, and the travel distance to the treatment centre can be burdensome for patients. Therefore, we developed “MS Fit”, a blended CBT for MS-related fatigue, based on a face-to-face CBT protocol found effective in a previous study, and “MS Stay Fit”, internet-based booster sessions to improve long-term effectiveness of CBT for MS-related fatigue. This article presents the protocol of two randomised clinical trials (RCTs) conducted within one study investigating (1) the non-inferiority of MS Fit compared with evidence-based face-to-face CBT for MS-related fatigue and (2) the effectiveness of MS Stay Fit on the long-term outcome of fatigue compared with no booster sessions.

**Methods/design:**

The first part of this study is an observer-blinded non-inferiority multicentre RCT, in which 166 severely fatigued MS patients will be randomly assigned (1:1 ratio, computer-generated sequence) to either face-to-face CBT or blended CBT (MS Fit) for fatigue. The primary endpoint is at 20 weeks after baseline. After this post-treatment assessment, patients will be randomly assigned again (1:1 ratio, computer generated sequence) to either MS Stay Fit consisting of two booster sessions at 2 and 4 months after end of treatment or no booster sessions. The primary endpoint of the second study is 52 weeks after baseline. Primary outcome measure in both studies is fatigue severity assessed with the fatigue severity subscale of the Checklist Individual Strength (CIS20r). Outcomes will be assessed at baseline (T0), at the end of treatment (T20), and after 39 and 52 weeks (T39 and T52).

**Discussion:**

If MS Fit is found to be non-inferior to face-to-face CBT, it will improve the accessibility of this treatment. In addition, the study aims to test whether it is possible to improve long-term effectiveness of CBT for MS-related fatigue with MS Stay Fit.

**Trial registration:**

Dutch Trial Register (NTR6966), registered 18 January 2018 https://www.trialregister.nl/trial/6782

**World Health Organization (WHO) Trial Registration Data Set:**

All items from the WHO Trial Registration Data Set can be found within the protocol.

## Background

Multiple sclerosis (MS) is a neurodegenerative disease characterized by demyelination, axonal loss and inflammation of the central nervous system that usually appears between the age of 20 and 40. MS can cause a variety of symptoms such as motor weakness, sensory deficits, impaired balance, fatigue, depression and pain. Fatigue is one of the most often reported symptoms in MS (75–90%) [1–4], and half of the patients with MS even report it to be one of the most burdensome symptoms affecting their daily functioning and worsening other MS symptoms [5].

The aetiology of MS-related fatigue is not clear and is likely to be multifactorial. In a cognitive-behavioural model, van Kessel and Moss-Morris proposed that where disease-related factors, such as inflammation and neurodegeneration, may initially cause fatigue, psychological mechanisms play an important role in perpetuating and increasing fatigue [6]. These mechanisms consist of cognitive, emotional and behavioural responses to fatigue (for example, catastrophizing cognitions, depression, helplessness, all-or-nothing behaviour, avoidance, and sleep problems). Based on this model, a cognitive behavioural therapy (CBT) was developed, aimed at decreasing fatigue, in which patients learn to change unhelpful beliefs and behaviours regarding fatigue [7, 8].

Although in clinical practice treatments for MS-related fatigue often include energy conservation management, exercise therapy, and multidisciplinary interventions, recent systematic reviews and meta-analyses of randomised clinical trials (RCTs) have shown CBT to be an effective treatment for reducing fatigue. There is no evidence for effectiveness of pharmaceuticals or multidisciplinary rehabilitation treatment [9–12]. In the recent TREFAMS-ACE programme, TREFAMS being an acronym for TReating FAtigue in Multiple Sclerosis, three RCTs were conducted studying the effectiveness of respectively Aerobic Exercise Training (TREFAMS-AT), Cognitive Behavioural Therapy (TREFAMS-CBT) and individual Energy Conservation Management (TREFAMS-ECM), compared with MS nurse consultations on fatigue [13]. Results showed that aerobic exercise training and energy conservation management did not lead to a significant or clinically relevant reduction of fatigue [14, 15], whereas the TREFAMS-CBT trial showed that MS-related fatigue can be reduced significantly with individual face-to-face CBT carried out by trained psychologists [16], although 1-year follow-up measurements showed that the effect of CBT had worn off over time. More studies have shown that fatigue in MS can be treated effectively with CBT [8, 17–19]. However, CBT was applied in different forms and protocols (individual and group formats, internet-based or face-to-face), and long-term effects differed between studies [20]. Analysis of cognitive-behavioural variables mediating the fatigue relapse in the TREFAMS-CBT study showed that increased daytime sleepiness, more problems in staying physically active and an increase of subjective cognitive problems (i.e., concentration) mediated the fatigue relapse after 1 year [7]. However, in these analyses, only a limited number of possible mediating factors were included. More research is needed to determine which other factors are responsible for the increase in fatigue at long-term follow-up.

Face-to-face CBT draws heavily on limited treatment capacity and requires patients to travel and schedule appointments which is burdensome for fatigued patients. Therefore, blended CBT, in which online treatment is combined with limited face-to-face contacts or video consultations with the CBT therapist, might be an attractive treatment option for severely fatigued patients with MS. Several studies have been conducted on the effectiveness of internet-based CBT for MS-related fatigue, showing positive results [17, 18, 21]. However, it remains unclear whether the effectiveness of blended CBT is comparable to that of face-to-face CBT in treating primary MS-related fatigue.

In the proposed study, two RCTs will be conducted to answer the following two research questions:

1. Is blended CBT (MS Fit) non-inferior with respect to its effect on fatigue severity compared with evidence-based face-to-face CBT in severely fatigued patients with MS?
2. Do blended CBT booster sessions (MS Stay Fit) improve long-term outcome with respect to fatigue severity at 1-year follow-up compared with no booster sessions?

To this purpose, “MS Fit”, an internet-based version of the CBT intervention used in the TREFAMS study, was developed [16]. In the MS Fit study group, patients will receive two face-to-face sessions with a CBT therapist and will be supported during their online treatment by video consultations and email contact. The online sessions of MS Fit are also based on the online CBT previously found to be effective in treating severe fatigue in patients with type 1 diabetes mellitus [22], breast cancer survivors [23] and patients with chronic fatigue syndrome [24].

In addition, “MS Stay Fit”, a blended booster programme consisting of several optional online booster modules and two video consultations with the therapist 2 and 4 months after finishing the initial CBT, has been developed. To our knowledge, this will be the first study testing the effectiveness of booster sessions in sustaining the effects of CBT for chronic fatigue. In this article, the SPIRIT (Standard Protocol Items: Recommendations for Interventional Trials) reporting guideline is used [25].

## Method/design

**Fig. 1:**
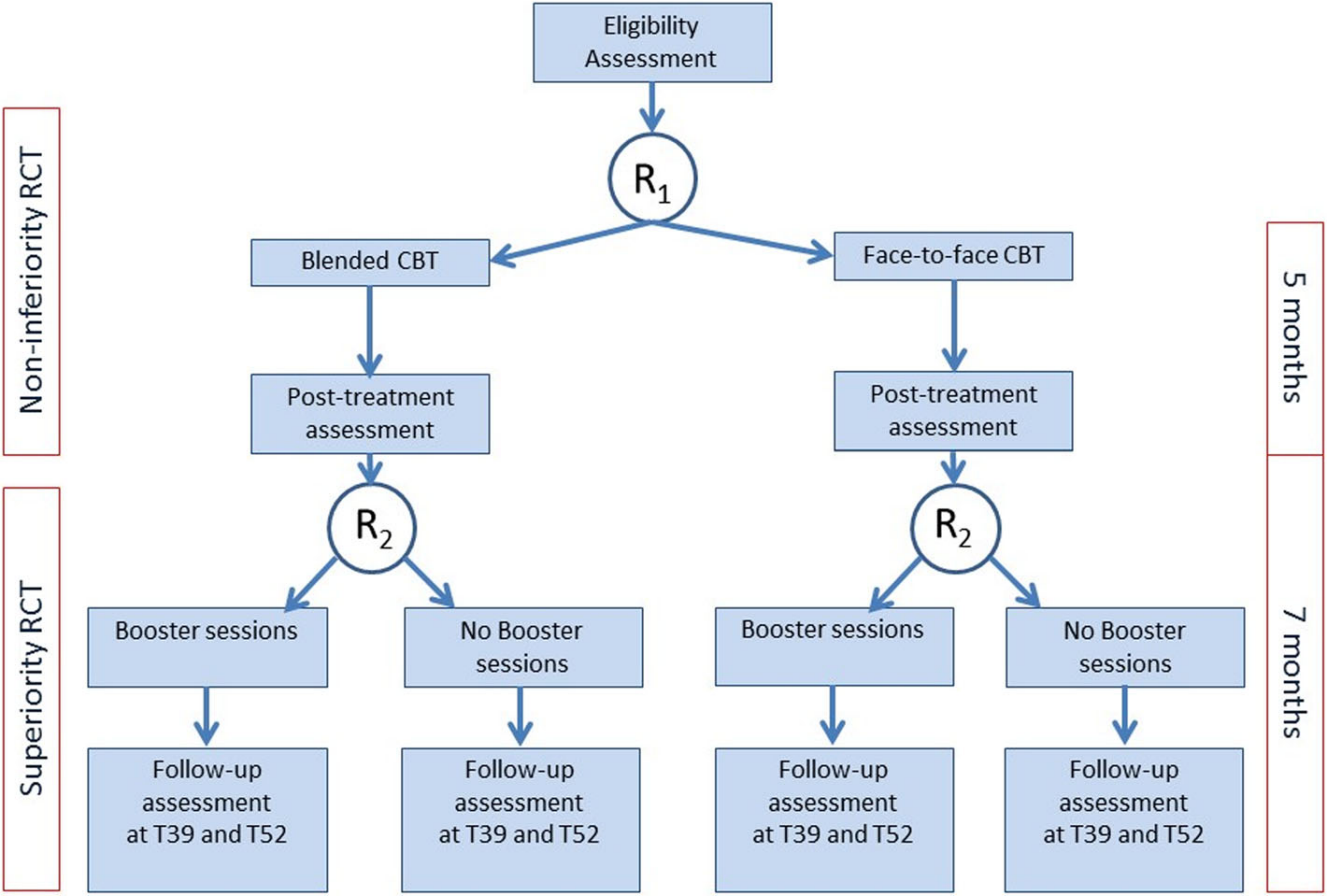
Study design

The first part of the study is an observer-blinded non-inferiority multicentre RCT, in which patients who are eligible to participate will be randomly allocated to either the face-to-face CBT intervention or MS Fit [26, 27]. Both groups will receive CBT for 20 weeks, and after a post-treatment assessment, patients who complete the second assessment will be randomly assigned again to MS Stay Fit or receiving no booster sessions. Patients who drop out of the initial treatment or study will not be randomly assigned for the second RCT. All patients will be assessed at baseline (T0), post-treatment (T20), after 9 months (T39) and after 1 year (T52) (Fig.1). See Additional file 1: Table S1 for all items of the World Health Organization (WHO) Trial Registration Data Set.

### Randomisation

Randomisation with concealed treatment allocation will be carried out by using a web-based randomisation facility. The randomisation scheme is computer-generated with stratification for treatment centre and using random variable block sizes (range 2–6). Using the same software, patients will be randomly assigned again (R2) at the end of the initial treatment to either MS Stay Fit or no booster sessions. Patients will be stratified according to previous CBT study group (Fig.1). Randomisation will be carried out by one of the project members (HB) who is not involved in the enrolment process, treatments or assessments.

### Participants

The 166 patients required for the non-inferiority RCT will have to meet the same inclusion criteria as in the TREFAMS-CBT study [13, 26, 27]: (a) definitive diagnosis of MS, (b) severely fatigued, (c) age of between 18 and 70, (d) ambulatory, (e) no evident signs of an exacerbation and no corticosteroid treatment in the past 3 months, (f) no current infections, (g) no anaemia, and (h) a normal thyroid function. The exclusion criteria are (a) depression, (b) primary sleep disorders, (c) other severe somatic or psychiatric co-morbidity, (d) current pregnancy or having given birth in the past 3 months, (e) pharmacological treatment for fatigue that was started in the past 3 months, (f) non-pharmacological therapies for fatigue that took place in the last 3 months, (g) having received CBT in the TREFAMS trial. This last exclusion criterion is added for this study. See Table 1 for the operationalization of the inclusion and exclusion criteria. As effectiveness of the booster programme can be measured only if patients have completed the initial treatment, the following additional inclusion criteria were defined for participation in the second randomized clinical trial testing the effectiveness of MS Stay Fit: (a) having started treatment (i.e., formulated treatment goals after the first session of CBT or MS Fit); (b) having finished the initial treatment period with a face-to-face session and assessment at T20.

**Table 1:** Inclusion and exclusion criteria of the non-inferiority trial

Inclusion criteria	Exclusion criteria
a. Definitive diagnosis of MS confirmed by a neurologist	a. Depression (BDI >4 and meeting criteria of depression as assessed by MINI)
b. Severely fatigued (CIS20r fatigue ≥35)	b. Primary sleep disorders (anamnesis)
c. Age between 18 and 70	c. Severe co-morbidity (CIRS item ≥3)
d. Ambulatory patients (EDSS ≤6)	d. Current pregnancy or having given birth in the past 3 months
e. No evident signs of an exacerbation, or corticosteroid treatment in the past three months	e. Pharmacological treatment for fatigue that was started in the past 3 months
f. No current infections (anamnesis)	f. Non-pharmacological therapies for fatigue that took place in the last 3 months
g. No anaemia (anamnesis)	g. Receiving CBT in the TREFAMS-CBT trial
h. A normal thyroid function (anamnesis)	

*Abbreviations*: *BDI* Beck Depression Inventory, *CBT* cognitive behavioural therapy, *CIRS* Cumulative Illness Rating Scale, *CIS20r* Checklist Individual Strength, *EDSS* Expanded Disability Status Scale, *MINI* mini international neuropsychiatric interview, *MS* multiple sclerosis, *TREFAMS* TREating FAtigue in MS

### Sample size calculation

#### Non-inferiority trial (RCT 1)

If there is truly no difference between the face-to-face CBT and blended CBT, then 150 patients are required to be 80% sure that the lower limit of a one-sided 95% confidence interval (CI) will be above the non-inferiority margin of −5.3 points on the Checklist Individual Strength (CIS20r) fatigue severity subscale. Adjusting for a dropout of 10%, 166 participants need to be included (two groups of 83) [26–28]. The non-inferiority margin was defined on the basis of statistical reasoning (i.e., by using the TREFAMS-CBT results) and clinical judgement [16]. In the TREFAMS-CBT trial, the difference in the change score between CBT and MS nurse consultations for fatigue was 6.67 points (95% CI 2.70–10.68) on the CIS20r fatigue severity subscale [16]. In accordance with the recommendations of the US Food and Drug Administration [26], the following formula was used to derive the margin of non-inferiority: the mean effect 6.67 - 50% of the lower limit of the 95% CI of the TREFAMS-CBT effect at week 16 (i.e. 2.7), that is, 6.67–1.35 = 5.32. A 1.35 point loss in effectiveness of blended CBT compared with TREFAMS-CBT is considered as clinically acceptable [16]. Tummers et al. [28] derived the same non-inferiority margin of 5.3 points on the CIS20r fatigue severity subscale through post-hoc analysis of the course in the waiting list of an RCT testing the efficacy of CBT in patients with chronic fatigue syndrome. The margin of 5.3 points reflects the largest loss of effect in the blended CBT group that would be clinically acceptable.

#### Booster trial (RCT 2)

This RCT concerns a superiority between-group comparison at 1-year follow-up of MS Stay Fit (i.e., web-based booster sessions combined with email contact and video consultations versus no booster sessions). Treatment effects on fatigue are assumed to be maintained with additional booster sessions but to gradually wear off to the fatigue level at baseline in the study group without additional booster therapy sessions. In the TREFAMS-CBT study, the treatment effects gradually wore off directly post-treatment until 1 year after the start of treatment [16]. At 1-year follow-up, fatigue levels in the CBT group were comparable to the level of fatigue at baseline. A between-group difference of 6.7 points on CIS20r fatigue is expected. Two-sided significance testing with an α of 5%, two study groups of 75 participants at 1 year, will result in a power of 98%. Therefore, the total number of enrolled patients (two groups of 83, i.e., 75 patients plus a 10% attrition rate) yields sufficient power in RCT 2 to address the second research question.

### Recruitment strategy

Patients will be recruited in 11 participating rehabilitation centres and hospitals in the Netherlands and one in Belgium (Table 2). More treatment sites can be added during the study if not enough eligible patients are available. Rehabilitation physicians will hand over the information letter to patients who are eligible for the study. When interested, patients will be contacted by phone by the research assistant to further inform them about the study. When willing to participate, they will be sent the informed consent form (ICF, a Dutch version is added as Additional file 3) in twofold to their home address and asked to return the signed ICF. Subsequently, patients are invited by email, sent by the research assistant, to complete the fatigue subscale of the CIS20r and the Beck Depression Inventory-Primary Care version (BDI-PC) online, after which all inclusion and exclusion criteria will be assessed in a phone call with the patient by the researcher. This includes assessment of the Expanded Disability Status Scale (EDSS) and Cumulative Illness Rating Scale (CIRS).

**Table 2:** Participating treatment centres

Amsterdam University Medical Centers, VU University Medical Center in Amsterdam	
Expert Center for Chronic Fatigue, Amsterdam University Medical Centers	
Maasstad Hospital in Rotterdam	
Rijndam rehabilitation center in Rotterdam	
Basalt rehabilitation center in Leiden	
Sint Maartenskliniek in Nijmegen	
Roessingh rehabilitation center in Enschede	
Klimmendaal rehabilitation center in Zutphen	
Canisius Wilhelmina hospital in Nijmegen	
Rehabilitation center Friesland in Sneek	
National MS center in Melsbroek (Belgium)	
Bravis hospital in Roosendaal and Bergen op Zoom	
Libra rehabilitation center in Tilburg	
De Hoogstraat rehabilitation center in Utrecht	

If patients contact the researcher directly and not via the rehabilitation physician (e.g., after reading about the study online), they will be referred for screening to a rehabilitation physician participating in this study before final inclusion and referral can take place.

### Interventions

#### Face-to-face cognitive behavioural therapy

In the face-to-face CBT study arm, patients receive 12 individual, face-to-face, 45-min therapy sessions distributed over a 20-week period in accordance with the protocol tested in the TREFAMS-CBT study [16]. CBT will be provided by certified health-care psychologists, most of them licensed cognitive behavioural therapists, who will be trained to deliver (blended) CBT for MS-related fatigue and receive supervision every two weeks.

#### Patient tailoring of (blended) CBT

CBT for MS-related fatigue is directed at the fatigue-maintaining behaviours and beliefs of the patient. The general aim of the CBT is to lessen the fatigue by changing fatigue-maintaining cognitions and behaviours and improve daily functioning. As the individual differences in the maintaining factors are substantial, first the relevant fatigue-maintaining factors for the individual patient are assessed by using specific screening instruments (Table 3). The patient is treated with the CBT modules aimed at the factors maintaining the fatigue of the individual patient. There are 10 treatment modules: (1) formulation and attainment of treatment goals, (2) sleep and rest, (3) unhelpful beliefs about MS, (4) beliefs about fatigue, (5) focusing on fatigue, (6) physical activity regulation, (7) regulation of social activity, (8) regulation of mental activity, (9) social support, and (10) unhelpful beliefs about pain. Therapists will get the results of the assessment to determine which CBT treatment modules are indicated for the individual patient. The following instruments are used for tailoring the CBT modules: Impact of Event Scale [31], Illness Cognition Questionnaire [32], Cognitive-Behavioural Responses to Symptoms [36, 37], Beck Depression Inventory – primary care version [33], Fear of Disease Progression-Short Form [34, 35], Self-Efficacy Scale [49], Jacobsen Fatigue Catastrophizing Scale [44], Illness Management Questionnaire [45], Social Support List (SSL and SSL D) [47] and Pain Catastrophizing Scale [48].

**Table 3:** Cognitive behavioural therapy modules and assessment tools used for patient tailoring of fatigue treatment

Treatment modules	Questionnaires and instruments
*1. Treatment goals*. Positive and concrete goals of the fatigue treatment are formulated by each patient. The goals consist of activities they would do when no longer severely fatigued.	All patients
*2. Sleep and rest*. The importance of a regular sleep–wake cycle and a good sleep hygiene are discussed, and instructions are given how to improve this.	Sickness Impact Profile subscale sleep and rest (scores ≥60) [29, 30] Sleep log during one week
*3. Uncertainty about the (consequences of the) illness and appraisal of MS as threatening*. In case of non-accepting cognitions of having MS and extreme fear of the future, the patient will be helped to gather realistic information about MS, to develop helping cognitions about MS and the personal future and to develop and maintain a more accepting attitude towards the illness and its consequences.	Impact Event Scale (IES ≥20) [31], subscale Acceptance of the Illness Cognition Questionnaire (ICQ-acceptance ≤12) [32], Beck Depression Inventory-PC (>4) [33], Fear of Disease progression Questionnaire–short form (FoP-Q-SF ≥34) [34, 35], The Cognitive behavioural Responses to Symptoms Questionnaire (CBRSQ) [36, 37]: - Resting behaviour >14.3, - all-or-nothing behaviour >12.9, - symptom focusing >15.5, - Embarrassment >16.4, - Damage >20.5, - Fear avoidance >15.3
*4. Fatigue-related cognitions*. Sense of control over fatigue symptoms (self-efficacy), fatigue catastrophizing, somatic attributions and other dysfunctional thoughts are assessed [38–43]. Patients are helped to change these cognitions in daily life.	modified Self Efficacy Scale for fatigue (≤19), Jacobson-Fatigue Catastrophizing Scale (≥16) [44]
*5. Focusing on fatigue*. Information about and consequences of focusing on fatigue will be discussed. Patients will practice with redirecting the focus of attention (away from the fatigue towards activity and other sensations).	Illness Management Questionnaire (≥4) [45]
*6. Physical activity regulation*. Depending on the activity pattern, patients will learn to spread activities more evenly, sometimes to lower activities and followed by a systematical increase of regular physical activity. After patients have increased their physical activity level, they increase other activities in order to reach the goals step by step.	Activity Pattern Interview
*7. Regulation of social activity*. The relationship with reduction in social activities as well as the cognitions about these activities and fatigue will be assessed in relation to the set goals. Suggestions how to increase social activities and how to handle the problems that are experienced during social interactions (as a consequence of cognitive impairments or intolerance of noise) are given.	Sickness Impact Profile (≥100) [29] subscale social functioning of the SF-36 (≤65) [46]
*8. Regulation of mental activity*. Patients are supported with regard to practicing and expanding mental activities such as computer use of reading. They learn how to deal with possible cognitive deficits such as concentration and memory problems.	CIS20r concentration subscale (score ≥18) [38]
*9. Social support*. The goal of this module is to support emotional independence of others as far as fatigue is concerned. Unrealistic expectations of others and expressing boundaries are discussed.	The Sonderen Social Support Inventory: subscale discrepancy (score ≥50) subscale negative interactions (score ≥14) [47]
*10. Unhelpful thoughts about pain*. Dysfunctional pain cognitions are challenged, and more helpful pain cognitions will be installed.	SF-36 bodily pain subscale (score ≤40) Pain Catastrophizing Scale (score ≥16) [48]

*Abbreviations: CIS20r* Checklist Individual Strength, *SF-36* 36-Item Short Form Survey

#### Blended CBT

Blended CBT (MS Fit) consists of two face-to-face contacts (one at the beginning and one at the end of the initial 20-week treatment period), three video consultations, and seven web-based therapy sessions with information and assignments delivered via an internet portal and supported by email contact with the therapist who provides feedback on the progress made by the patient. The blended CBT will also be patient-tailored on the basis of the same 10 treatment modules described earlier (Table 3). The therapists who deliver the face-to-face CBT will also deliver MS Fit, and the treatments are supervised once every two weeks.

#### Development of MS Fit and usability testing

The information and assignments provided in the internet portal are developed by experts on CBT for MS-related fatigue and are based on “Dia-Fit”, an evidence-based blended CBT intervention for severely fatigued patients with diabetes type 1 [22], and “On the road to recovery”, an evidence-based intervention for cancer-related fatigue in breast cancer survivors [23]. The interventions were adapted for MS. Comparable treatment modules are formulation of treatment goals, sleep and rest, beliefs about fatigue, focusing on fatigue, and regulation of activity (Table 3). Two specific modules about MS (namely unhelpful beliefs about MS and pain) were developed by experts on chronic fatigue and MS. Two patients with MS who already had received face-to-face CBT for fatigue and one patient with MS who participates in the trial steering committee on behalf of the Dutch patient organisation MSVN were asked for usability testing of the portal. Their feedback was used to improve parts of the intervention.

#### MS Stay Fit

The booster programme MS Stay Fit consists of two patient–therapist booster consults with the same therapist who delivered the first part of the intervention, scheduled at 2 and 4 months after the end of initial treatment. Booster sessions will be delivered via the internet via a secured video connection and combined with email contact and web-based CBT assignments on prevention of fatigue relapses. Five optional “booster modules” are aimed at the factors that mediated the relapse in fatigue following the intervention in the original trial: sleep–wake pattern and activity regulation (both physical and mental) [7] but also at cognitions about fatigue, coping with “normal fatigue” and continuing reaching the set goals.

### Therapist training and supervision

All involved therapists received a 3-day training course in the face-to-face CBT protocol for MS-related fatigue and a 1-day training course in delivering the e-health intervention. The CBT training course consists of training the content and indication of each treatment module and practicing the intervention techniques in role-playing with help of professional actors who simulate patients. The e-health training course consists of an introduction to the online platform, practicing in delivering MS Fit, and skills in writing emails. All therapists will provide both face-to-face and blended CBT to make sure that any between-group differences found are not contributable to specific therapist factors or treatment centres. All therapists will receive bi-weekly supervision via the telephone by a psychologist with extensive experience in the treatment of chronic fatigue (MG and HK).

### Modification of allocated intervention

Reasons for discontinuing the allocated intervention can be new health problems or life events hindering patients to continue the treatment or patient’s decision to withdraw from the treatment.

After 8 months, a small adjustment was made in the introduction page of MS Fit, after receiving feedback of two patients who found the description not applicable to them, demotivating them to continue treatment. These sentences were adjusted, and an example was added, in order to clarify the purpose of the blended CBT.

### Concomitant care

Patients are instructed not to follow or use any other therapies or medication aimed at fatigue during the 1-year study period. Multidisciplinary rehabilitation treatments aimed at other health problems should be phased before or postponed after the 20-week CBT intervention. All other medical interventions are allowed. Use of additional fatigue management is not systematically registered, since it would be difficult to reliably assess this during the course of 1 year, and the assumption is made that this will be comparable between all groups.

### Outcome measures

**Fig. 2:**
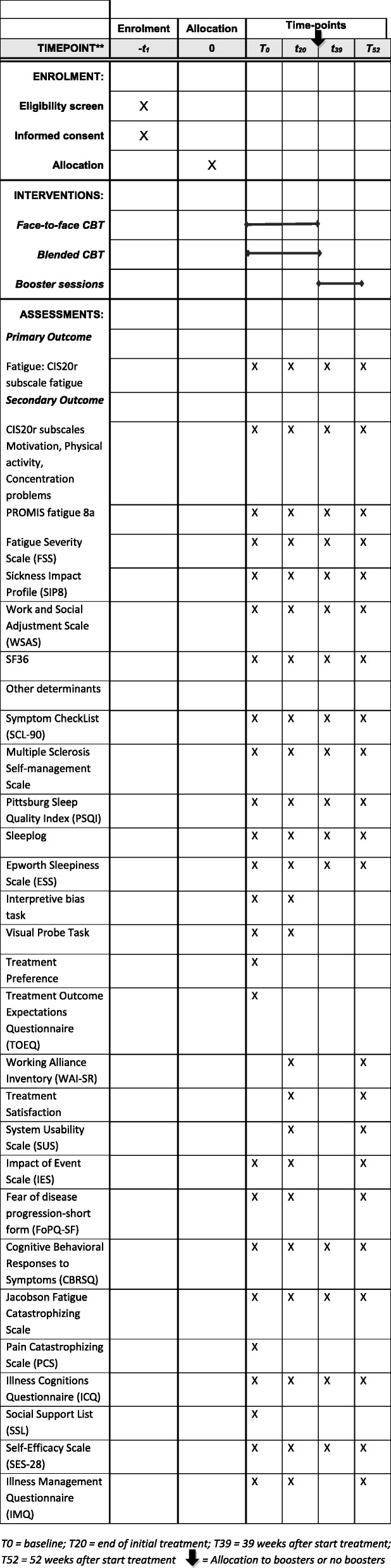
Time points of all measurements

Outcome measures consist of validated self-reported questionnaires, which will be assessed at baseline (T0); the end of initial treatment (T20), which is equal to the end of the non-inferiority RCT 1; and at 39 and 52 weeks (end of RCT 2). See Fig. 2 for measurements at all time points. Patients can complete these questionnaires online from their home. At all these time points, for 1 week, patients will also keep an online sleep log, which is sent to them daily for 7 consecutive days. Completing the questionnaires online requires filling in every item, which prevents occurrence of missing item values. The research assistant checks for possible missing sleep logs, and, if necessary, sends additional sleep logs as a reminder to the patient.

#### Primary outcome measure

In both RCTs, the primary outcome measure will be fatigue severity, as measured by the CIS20r fatigue severity subscale, either at the end of initial treatment (RCT 1: T20) or at 1 year (RCT 2: T52). At these time points, the differences in fatigue severity between both groups will be assessed. The same primary outcome measure is used as in the TREFAMS-CBT trial. The CIS20r is a 20-item self-reported questionnaire consisting of four subscales: (a) fatigue severity (eight items), (b) reduction in motivation (four items), (c) reduction in physical activity (three items) and (d) concentration problems (five items) [38, 50]. Items are answered on a 7-point Likert scale and added to a total score. The total score of the fatigue severity subscale can vary between 8 and 56 points. The CIS20r is a reliable and valid instrument for measuring fatigue in various study populations [39]. A cutoff score of 35 or higher is used to discriminate between severe fatigue and fatigue levels within the normal range.

#### Secondary outcome measures

Clinical significant improvement assessed with the CIS20r is a secondary outcome measure. Clinical significant improvement is defined as either a score of less than 35 on the CIS20r fatigue severity subscale or an improvement of at least 8 points on this subscale [16]. As the CIS20r is also used as a screening tool thereby limiting the variance in the outcome measure, we decided to use the following additional fatigue measures as secondary outcome measures. The Fatigue Severity Scale (FSS) is used to measure the severity and impact of fatigue in patients with MS [51]. The FSS consists of nine items to be rated on a scale from 1 (strongly disagree) to 7 (strongly agree). The final score is the mean of all item scores. Also, the PROMIS-Fatigue Short Form 8a [52] is used to measure fatigue in addition to the CIS20r. This questionnaire consists of eight items to be rated on a scale from 1 (not at all/never) to 5 (very much/always). The final score is the sum score of all items.

The Sickness Impact Profile (SIP) [29, 30] will be used to measure limitations of daily functioning in eight different domains of functioning. The eight subscale scores are summed to provide one weighted score of disability (SIP8 total score). The SIP was used in several intervention studies with chronically fatigued patients. The Work and Social Adjustment Scale (WSAS) [53] is used to measure restrictions in participation. The WSAS is a five-item scale rating impairment on five different domains of functioning on a scale from 1 (not at all impaired) to 8 (severely impaired). The final score is the sum of all items. Health-related quality of life will be assessed by the SF36 [46], consisting of 36 items and eight subscales. Raw scores on each scale are converted to a score on a scale of 0 to 100, and a higher score indicates a higher level of well-being.

For these secondary outcome measures, no margins of non-inferiority have been defined. The course of the scores on these secondary outcome measures during the whole study year will be plotted for the various subgroups of participants. See Additional file 2: Table S2 for specifications of the primary and secondary outcome measures.

#### Demographic and disease characteristics

The demographic characteristics that will be assessed are age, gender, ethnicity, level of education and employment. Disease-related determinants that are assessed include self-reported type of MS, date of diagnosis, date of onset of MS, use of disease-modifying drugs, and the score on the EDSS [54] and comorbidities using the CIRS [55], both assessed by the researcher at baseline.

#### Moderators and mediators

Since various factors may play a moderating or perpetuating role in fatigue and since CBT consists of multiple modules that intervene on different aspects of fatigue, it is interesting to measure variables that (1) may moderate the effect of blended CBT in comparison with face-to-face CBT and/or (2) moderate or mediate the effects of the booster sessions of CBT on long-term outcome [7, 37]. In addition to the measurement instruments used to tailor the CBT modules, the following factors will be assessed at several time points for the analysis of moderation and mediation. Psychological distress will be assessed by using the total score on the Symptom Check List (SCL-90) [56, 57]. Daytime sleepiness will be assessed by the Epworth Sleepiness Scale [58]. Sleep dysfunction is assessed by the Pittsburgh Sleep Quality Index [59]. A sleep log of 1 week will be used to assess sleep parameters. Self-management will be measured with the Multiple Sclerosis Self-Management Scale [60].

##### Attentional and Interpretative Bias

To measure the tendency of patients to direct their attention to illness-related information, a Visual Probe Task (VPT) is used. This task was developed by Hughes et al. [61] to measure an attentional bias for illness-related information in patients who are chronically fatigued. As this computer task is programmed using e-Prime, it needs to be performed on a computer under controlled conditions. Therefore, only participants living in the Amsterdam area will be asked to complete this task. The VPT will be assessed prior to the first and last treatment session at the treatment centre by a research assistant. Furthermore, an interpretive bias task will be used to assess the tendency of patients to interpret bodily sensations in a negative, threatening way [61]. We aim to assess patients before and after treatment (T20) and to determine whether a change in the tendency of patients to focus on symptoms is related to the reduction in fatigue brought on by CBT.

##### Treatment preference, outcome expectation and treatment satisfaction

Patient–therapist factors will be taken into account by assessing treatment preference (online vs. face-to-face CBT), and patients expectations of treatment outcome using the Treatment Outcome Expectations Questionnaire (TOEQ) [62] at baseline. At the end of treatment (T20) and 1 year after the start of treatment (T52), therapeutic alliance will be assessed by using the Working Alliance Inventory (WAI-SR) [63, 64]. Also, patients’ satisfaction with treatment and satisfaction with the platform will be assessed by using the System Usability Scale (SUS) [65, 66].

### Participant retention

Participants receive the questionnaires online. For optimal retention, participants will be contacted by email or phone by the research assistant when they have not started the completion of the questionnaires within 1 week. Randomization is carried out after completing all questionnaires at T0 (R1) or T20 (R2), preventing missing data at these important moments. When participants are not willing to complete all measurements at T20, T39 or T52, they are asked to at least fill in the fatigue subscale of the CIS20r, which the primary outcome measure.

### Participant withdrawal

Patients can leave the study at any time for any reason if they wish to do so without any consequences. Nevertheless, these patients are requested to fill in a final CIS20r fatigue. The researcher can withdraw a patient from the study in case of incorrect enrolment of the participant. Withdrawing from the study does not necessarily mean the patient has to stop treatment. The treating physician or the treating psychologist can decide to withdraw a patient from the study for urgent (medical) reasons. These reasons will be documented.

### Serious adverse events

If patients or therapists report adverse events, these will be recorded in the case record form. CBTs, both face-to-face and blended, are expected to be safe treatment methods. However, all serious adverse events (SAEs) will be reported in accordance with the Dutch Act on Medical Research Involving Human Subjects. An SAE is defined as any untoward medical occurrence or effect that is lethal or life-threatening (or both), requires hospitalisation or prolongation of existing inpatients’ hospitalization, or results in persistent or significant disability or incapacity. SAEs will be reported to the researcher by the therapists during by-weekly supervision or by email. All SAEs need to be reported in a timely fashion to the Medical Ethics Review Committee. When adverse events occur, appropriate diagnostic procedures and medical treatment will be undertaken as needed. The ethics committee has granted dispensation for insurance for damage to research participants through injury or death caused by the study, as participation in this study is without risks.

### Treatment fidelity and compliance

After the first therapy session, each therapist registers which modules are indicated based on the patient-reported questionnaires and which modules are indicated based on their interview. With regard to the process, they will register each provided therapy session or patient contact (face-to-face, video, phone, email), the duration of the contact and which of the indicated treatment modules were delivered in that session. Patient therapy adherence in MS Fit will be assessed via log data provided by the online platform. All therapists will be supervised bi-weekly by an experienced clinical psychologist (HK or MH) to ensure patient-specific treatment integrity.

### Blinding of outcome measurements and data analysis

In this study, mainly patient-reported outcomes will be gathered. Patients and therapists cannot be blinded for the type of treatment. A research assistant, who will be blinded with respect to treatment allocation, will send out the internet questionnaires and will monitor the timely completion of filling in the questionnaires. The statistical analyses will be carried out by an independent researcher on an encrypted data file, blinded for treatment allocation.

### Handling and storage of data and documents

Data, other than the questionnaires, are entered in an electronic case report file (eCRF), which includes an audit trail. Personal data will be handled confidentially and in a coded way and will comply with the Dutch Personal Data Protection Act. Patient identification will be coded for all study procedures. Only the project leader and primary researcher have access to the codes and participant data. Codes and participant data will be stored in password-protected files. After finishing the study, the key to the code will be safeguarded by the coordinating investigator. Data will be stored by the Department of Rehabilitation Medicine of the Amsterdam University Medical Centers for 15 years following completion of the project.

Therapist secrecy and confidentiality will be maintained at all times. Patient correspondence by emails will be encrypted and securely stored to guarantee privacy and confidentiality. An email account of the university hospital will be used for correspondence by emails. Participant information will not be disclosed to third parties. Only the trial steering committee will have access to the full dataset.

### Biological specimens

No biological specimens will be collected in the study.

### Monitoring and auditing

This study will be subject to on-site monitoring in accordance with the quality assurance advice of the Dutch Federation of University Medical Centres regarding research involving human subjects [67]. On-site monitoring will be based on the risk classification (negligible).

The sponsor/investigator will submit a summary of the progress of the trial to the ethics committee once a year. Information will be provided on the date of inclusion of the first subject, numbers of subjects included and numbers of subjects that have completed the trial, SAEs/serious adverse reactions, other problems, and amendments.

### Statistical analyses

#### Non-inferiority of blended CBT compared with face-to-face CBT at 20 weeks

**Fig. 3:**
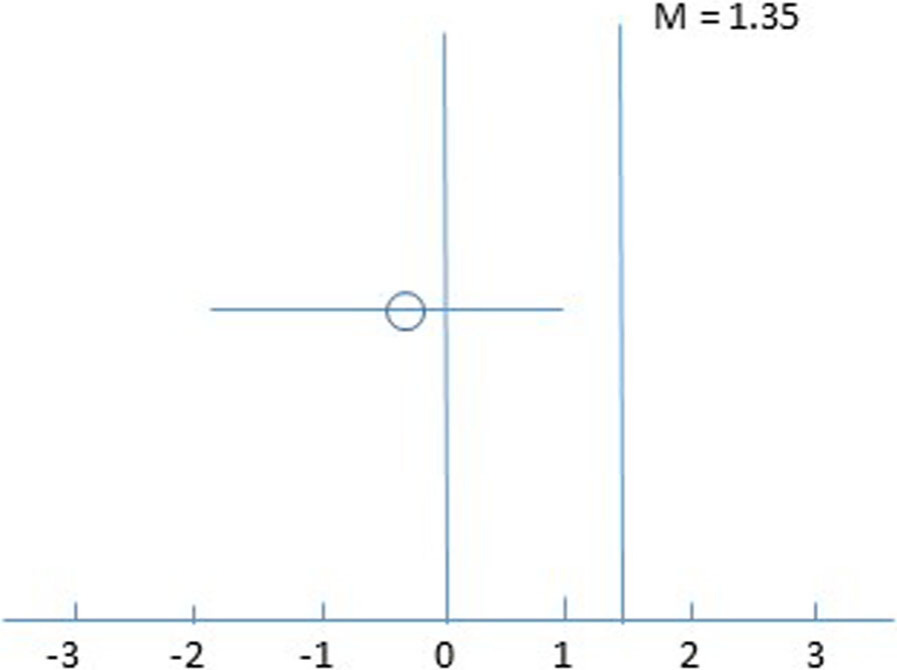
Illustration of a possible result of the non-inferiority tria. Difference between face-to-face CBT and blended CBT and the 95% CI. H0: blended CBT is inferior to face-to-face CBT, meaning that face-to-face CBT – blended CBT ≥ M. Mis the non-inferiority margin (6.67–5.32 = 1.35). H1: blended CBT is non-inferior to face-to-face CBT, meaning that face-to-face CBT – blended CBT < Ml

Primary analyses will be on the basis of intention-to-treat (ITT) and per protocol using mixed model analysis in order to reduce the risk of falsely claiming non-inferiority of blended CBT [26, 27]. These analyses will be performed with the CIS20r fatigue severity score at the end of treatment (T20) as dependent variable and study group (blended vs. f2f CBT) as fixed factor. A graph showing CIS20r fatigue CIs and margins of non-inferiority will be used (see Fig. 3) [26, 27]. When blended care is found to be non-inferior, a sensitivity analysis will be performed on the basis of different assumptions about the values of missing data.

To compare the effect of blended care and face-to-face CBT on the secondary outcome measures, the ITT mixed model analysis will be repeated with the secondary outcomes at the end of treatment (T20) as dependent variable and study group as fixed factor.

#### Effectiveness of CBT booster sessions at 52 weeks

Primary analysis of long-term effects of additional booster sessions will be based on ITT using mixed model analysis with CIS20r fatigue severity 1 year after the start of treatment (T52) as dependent measure and condition (booster session vs. no booster sessions) as fixed factor. In case of a large attrition rate, a per-protocol analysis will be added.

To compare the effect of booster sessions on the secondary outcome measures, the ITT mixed model analysis will be repeated with the secondary outcomes at T52 as dependent variable and study group as fixed factor.

In a separate analysis, we will additionally investigate the differential effect of the booster sessions in patients who are no longer severely fatigued at T20 compared with those who are still fatigued at T20.

Analyses of factors mediating long-term effectiveness will be conducted. In addition, analyses will be carried out to identify possible moderators of the response to the booster sessions and response to face-to-face or blended CBT.

## Discussion

A study protocol was presented of two RCTs combined in one study. First, non-inferiority will be tested of MS Fit (blended CBT) on fatigue severity compared with face-to-face CBT in severely fatigued patients with MS [13, 16]. Subsequently, superiority will be tested of MS Stay Fit, i.e., additional internet-based booster sessions following MS Fit or face-to-face CBT, compared with no booster sessions on fatigue severity up to 1 year after randomisation.

By combining two RCTs, this study is able to address two important research questions. The non-inferiority trial aims to give more insight in whether the CBT protocol applied in a blended form is non-inferior as to when applied face-to-face. If MS Fit is indeed non-inferior to face-to-face CBT, it probably will be a more time-efficient but also a more accessible treatment option, enhancing implementation of this intervention. Since long-term effects of CBT vary between studies with some showing considerable fatigue relapse [20] and since little is known about the underlying mechanisms of fatigue relapses after successful treatment [7], testing the effect of booster sessions and testing possible mediators and moderators of the response to treatment may contribute to a better understanding of these mechanisms and, most importantly, improving long-term effects. To the best of our knowledge, this is the first study testing the effectiveness of booster sessions in treating chronic severe fatigue. In addition, by training psychologists of 12 participating rehabilitation centres and hospitals, this study aids in implementation of CBT for MS-related fatigue in the Netherlands and Belgium, making effective fatigue treatment more accessible to patients with MS.

In this trial, inclusion criteria and the face-to-face CBT protocol used are similar as in the TREFAMS-CBT study. However, in our study, more rehabilitation centres are including and treating patients, and, even with the same set of enrolment criteria, this may lead to a more varied patient sample. Given the number of therapists (currently 20 therapists are involved) in the study, it is also plausible that there is more variance in therapist-related factors (e.g., familiarity with providing e-health interventions such as blended CBT). Providing an extensive manual, intensive training and supervision of all therapies intends to limit these variants and optimize treatment integrity as much as possible [68], respecting the consistency rules of non-inferiority trials [26, 27]. Taking this into account, these aspects may also well increase generalizability of the results of this pragmatic trial to clinical practice. In addition, in this trial, treatment duration is set at 20 weeks instead of 16 weeks since this is expected to increase opportunities for patients to reach treatment goals and cognitive and behavioural changes.

Non-inferiority of blended CBT is measured by means of fatigue severity as the primary outcome measure, but benefits of treatment may regard other aspects as well (e.g., accessibility, time-efficiency or adherence). Being effective but having a high dropout rate, for example, can also have implications for implementation. These factors will be measured and might help in interpreting the primary trial results.

Even though the content of treatment is similar in both treatments, differences in form of treatment may affect several factors possibly influencing effectiveness. It is known that factors in the therapy relationship (e.g., outcome expectations and working alliance) can contribute to treatment outcome in general [40] but also more specifically in treating fatigue [62]. In general, working alliance is found to be related to self-efficacy, outcome expectancies, treatment adherence and satisfaction [41]. Since in the blended-CBT group therapist contact is more distant and less frequent, this might influence treatment adherence, satisfaction and outcome effects. For that reason, patients’ evaluation of the working alliance, treatment satisfaction and usability of the platform will be taken into account in secondary trial analyses. Outcome expectations are also measured at baseline, but at the time of measurement, patients will not be randomly assigned yet, so these expectations will not apply to the form of treatment that they will adhere to. It could be argued that treatment expectations may be higher in the present study than in the TREFAMS-CBT study since CBT for MS-related fatigue is proven effective now.

In conclusion, this paper presents the study protocol of two combined RCTs testing non-inferiority of blended CBT compared with face-to-face CBT for MS-related fatigue, and the effect of booster sessions on long-term outcome. This study aims to provide more insight in effectiveness of CBT in various applied forms, and in possibilities for improving long-term effectiveness of the treatment. In addition, by conducting the study and training of psychologists in 12 rehabilitation centres in the Netherlands and Belgium, this study is a first step in implementation of CBT in clinical practice.

### Trial status

Patient recruitment started in April 2018 and is expected to end in March 2021. At the time of submission, 64 patients have been randomly assigned. The trial is being conducted in accordance with the protocol version 7, dated 4 July 2019.

## Supplementary information

**Additional file 1: Table S1.** World Health Organization (WHO) Trial Registration Data Set.

**Additional file 2:** **Table S2.** Specifications of outcome measures.

**Additional file 3.** Informed consent form (in Dutch).

## Data Availability

Any request to share the data of these RCTs will be considered by the trial steering committee and will need to be approved by the ethics committee of the Amsterdam University Medical Centers, VU University Medical Center, before granted. An informed consent form in Dutch is available as a supplement.

## Abbreviations

CBT: Cognitive behavioural therapy
CI: Confidence interval
CIRS: Cumulative Illness Rating Scale
CIS20r: Checklist Individual Strength
eCRF: Electronic case report file
EDSS: Expanded Disability Status Scale
FSS: Fatigue Severity Scale
ICF: Informed consent form
ITT: Intention-to-treat
MS: Multiple sclerosis
RCT: Randomized clinical trial
SAE: Serious adverse event
SIP: Sickness Impact Profile
TREFAMS: TREating FAtigue in MS
VPT: Visual Probe Task
WSAS: Work and Social Adjustment Scale
