# Supplementary material for: Testing non-inferiority of blended versus face-to-face cognitive behavioural therapy for severe fatigue in patients with multiple sclerosis and the effectiveness of blended booster sessions aimed at improving long-term outcome following both therapies: study protocol for two observer-blinded randomized clinical trials
Source: Trials. 2020 Jan 20;21:98. doi: 10.1186/s13063-019-3825-2 (PMC6971870; doi:10.1186/s13063-019-3825-2)
Supplement: Supplementary file 1 — Additional file 1: Table S1. World Health Organization (WHO) Trial Registration Data Set. [file 13063_2019_3825_MOESM1_ESM.docx]

Table S2 Specifications of outcome measures

|  | **Domain** | **Measure** | **Method of aggregation** | **Metric** | **Time point** |
| --- | --- | --- | --- | --- | --- |
| *Primary outcome measure* | Fatigue | CIS20r subscale fatigue severity | Sum score of 8 items in this subscale | Difference between groups at T20 and T52 | RCT 1: end of initial treatment (T20)  RCT 2: one year (T52) |
|  | | | |  |  |
| *Secondary outcome measures* | Fatigue | Fatigue Severity Subscale | Mean score of 9 items in this scale |  |  |
|  | Fatigue | PROMIS Fatigue- short form 8a | Sum score of 8 item in this scale |  |  |
|  | Fatigue | CIS20r subscale reduction in motivation  CIS20r subscale reduction in physical activity  CIS20r subscale concentration problems | Sum score of 4 items  Sum score of 3 items  Sum score of 5 items |  |  |
|  | Limitations in daily functioning | Sickness Impact Profile (SIP) | The scores on the 8 subscales are summed to provide one weighted score |  |  |
|  | Restrictions in participation | Work and Social Adjustment Scale (WSAS) | Sum score of 5 items. |  |  |
|  | Quality of life | SF36 | Sum of the weighed scores of 8 subscales |  |  |
