# Supplementary material for: Testing non-inferiority of blended versus face-to-face cognitive behavioural therapy for severe fatigue in patients with multiple sclerosis and the effectiveness of blended booster sessions aimed at improving long-term outcome following both therapies: study protocol for two observer-blinded randomized clinical trials
Source: Trials. 2020 Jan 20;21:98. doi: 10.1186/s13063-019-3825-2 (PMC6971870; doi:10.1186/s13063-019-3825-2)
Supplement: Supplementary file 2 — Additional file 2: Table S2. Specifications of outcome measures. [file 13063_2019_3825_MOESM2_ESM.docx]

Table S1 WHO Trial Registration Data Set

| Primary Registry and Trial Identifying Number | Dutch Trial Register (NTR)  NTR6966 |
| --- | --- |
| Date of Registration in Primary Registry | 19January 2018 |
| Secondary Identifying Numbers | NL62622.029.17 and 16-937 MS |
| Source(s) of Monetary or Material Support | [Dutch MS Research Foundation](http://www.trialregister.nl/trialreg/admin/rctsearch.asp?FC=970) (grant no: 16-937 MS) |
| Primary Sponsor | [Prof. dr. Vincent de Groot, Amsterdam UMC, VU University Medical Center Department of Rehabilitation Medicine](http://www.trialregister.nl/trialreg/admin/sponsorview.asp?SC=1662) |
| Secondary Sponsor(s) | Prof. dr. J.A. Knoop,  Amsterdam University Medical Centers, Amsterdam Medical Center, Department of Medical Psychology  dr. H. Beckerman, Amsterdam UMC, VU University Medical Center Department of Rehabilitation Medicine |
| Contact for Public Queries | M. Houniet-de Gier  Department of Medical Psychology  Amsterdam University Medical Centers (VUmc)  PO BOX 7057  1007 MB Amsterdam  The Netherlands  e-mail: [m.degier1@amsterdamumc.nl](mailto:m.degier1@amsterdamumc.nl)  phone: +31 20 4440136 |
| Contact for Scientific Queries | Principal Investigator:  Prof. dr. Vincent de Groot  Amsterdam UMC, location VU University Medical Center  Department of Rehabilitation Medicine  PO BOX 7057, 1007 MB Amsterdam  [v.degroot@amsterdamumc.nl](mailto:v.degroot@amsterdamumc.nl)  Contact for scientific queries:  M. Houniet-de Gier  Department of Medical Psychology  Amsterdam University Medical Centers (VUmc)  PO BOX 7057  1007 MB Amsterdam  The Netherlands  e-mail: [m.degier1@amsterdamumc.nl](mailto:m.degier1@amsterdamumc.nl)  phone: +31 20 4440136 |
| Public Title | e-TREFAMS-CBT: Blended CBT for MS-related fatigue |
| Scientific Title | Blended versus face-to-face Cognitive Behavioural Therapy in treating severe fatigue in patients with MS : an observer-blinded randomized clinical trial testing non-inferiority at post-treatment and long-term effectiveness. |
| Countries of Recruitment | The Netherlands  Belgium |
| Health Condition(s) or Problem(s) Studied | Multiple Sclerosis, Fatigue, [Cognitive behaviour therapy](http://www.trialregister.nl/trialreg/admin/rctsearch.asp?DC=692), [Multiple Sclerosis-related fatigue](http://www.trialregister.nl/trialreg/admin/rctsearch.asp?DC=5713), [Blended Cognitive Behavioural Therapy](http://www.trialregister.nl/trialreg/admin/rctsearch.asp?DC=5714) |
| Intervention(s) | TREATMENTS  First random allocation:  1.1 Face-to-face CBT (TREFAMS-CBT protocol)  1.2 Blended CBT Second random allocation:  2.1 Booster sessions 2.2 No booster sessions  The design and interventions are explained in the protocol. |
| Key Inclusion and Exclusion Criteria | Inclusion criteria of study participants:  a) definitive diagnosis of MS; b) severely fatigued (CIS20r fatigue =>35); c) ambulatory patients (EDSS <=6); d) no evident signs of an MS exacerbation, or a corticosteroid treatment in the past 3 months; e) no current infections; f) no anaemia; g) a normal thyroid function.  Exclusion criteria: a) depression (assessed with BDI-II-PC ≥ 4 and M.I.N.I) ; b) primary sleep disorders; c) severe comorbidity (CIRS item scores ≥ 3); d) current pregnancy or having given birth in the past 3 months; e) pharmacological treatment for fatigue that was started in the past 3 months (e.g. Amantadine, Modafinil, Ritalin, Pemoline); f) non-pharmacological therapies for fatigue that took place in the past 3 months. Patients who already received CBT in the TREFAMS study will be excluded as well. |
| Study Type | 1. Non-Inferiority Randomized clinical trial, , observer-blinded, blended CBT vs face-to-face CBT 2. Superiority RCT of boosters vs no boosters   Randomisation with concealed treatment allocation will be carried out using a web-based randomisation facility. The randomisation scheme is computer-generated with stratification for treatment centre, and using random variable block sizes (range 2-6). |
| Date of First Enrolment | April 2018 |
| Sample Size | Total sample size is based on the non-inferiority trial (RCT1): 166 patients. 150 patients are required to be 80% sure that the lower limit of a one-sided 95% confidence interval will be above the non-inferiority margin of -5.3 points on the Checklist Individual Strength (CIS20r) fatigue severity subscale. Adjusting for a drop-out of 10%, 166 participants need to be included (2 groups of 83). |
| Recruitment Status | Recruiting: participants are currently being recruited and enrolled. |
| Primary Outcome(s) | Outcome: **Fatigue severity**  Metric/method of measurement: Checklist Individual Strength, subscale fatigue  Time point: - RCT 1: End of treatment (week 20)  - RCT 2: one year after start of treatment (week 52) |
| Key Secondary Outcomes | Outcome: **Fatigue**  Metric/method of measurement: - PROMIS fatigue– short form 8a  - Fatigue Severity Scale  - Checklist Individual Strength, subscales  reduction in motivation due to fatigue,  reduction in physical activity due to  fatigue, and concentration problems  Time point: - RCT 1: End of treatment (week 20)  - RCT 2: one year after start of treatment (week 52)  Outcome: **Limitations of daily functioning**  Metric/method of measurement: Sickness Impact Profile  Time point: - RCT 1: End of treatment  - RCT 2: one year after start of treatment  Outcome: **Restrictions in participation**  Metric/method of measurement: Work and Social Adjustment Scale  Time point: - RCT 1: End of treatment  - RCT 2: one year after start of treatment  Outcome: **Health-related quality of life**  Metric/method of measurement: SF36  Time point: - RCT 1: End of treatment  - RCT 2: one year after start of treatment |
| Ethics Review | Approved by the Medical Ethical Committee of the Amsterdam University Medical Centers, location VU University Medical Center (registration number 2017.538, NL62622.029.17. In addition, all participating centers have issued a local consent statement. |
