# Supplementary material for: Testing non-inferiority of blended versus face-to-face cognitive behavioural therapy for severe fatigue in patients with multiple sclerosis and the effectiveness of blended booster sessions aimed at improving long-term outcome following both therapies: study protocol for two observer-blinded randomized clinical trials
Source: Trials. 2020 Jan 20;21:98. doi: 10.1186/s13063-019-3825-2 (PMC6971870; doi:10.1186/s13063-019-3825-2)
Supplement: Supplementary file 3 — Additional file 3. Informed consent form (in Dutch). [file 13063_2019_3825_MOESM3_ESM.docx]

Informed Consent Form (in Dutch)

**Toestemmingsverklaring**

**Internet-based versus face-to-face cognitieve gedragstherapie voor MS-gerelateerde vermoeidheid**

*Blended versus face-to-face cognitieve gedragstherapie als behandeling voor ernstige vermoeidheid bij patiënten met MS- een observer-blinded randomized controlled trial om non-inferioriteit en de lange termijn effecten te onderzoeken.*

- Ik heb de informatiebrief gelezen. Ook kon ik vragen stellen. Mijn vragen zijn voldoende beantwoord. Ik had genoeg tijd om te beslissen of ik meedoe.
- Ik weet dat meedoen vrijwillig is. Ook weet ik dat ik op ieder moment kan beslissen om toch niet mee te doen of te stoppen met het onderzoek. Daarvoor hoef ik geen reden te geven.
- Ik weet dat mijn huisarts wordt geïnformeerd dat ik meedoe aan dit onderzoek
- Ik weet dat sommige mensen mijn gegevens kunnen inzien. Die mensen staan vermeld in deze informatiebrief.
- Ik geef toestemming voor het verzamelen en gebruiken van mijn gegevens op de manier en voor de doelen die in de informatiebrief staan
- Ik geef toestemming om mijn gegevens op de onderzoekslocatie nog 15 jaar na dit onderzoek te bewaren.
- Ik geef □ **wel**

□ **geen** toestemming om mij na dit onderzoek opnieuw te benaderen voor een vervolgonderzoek.

- Ik geef □ **wel**

□ **geen** toestemming om mijn gegevens eventueel te gebruiken voor extra onderzoek

- Ik geef toestemming aan de VUmc onderzoeker, Marieke Houniet- de Gier, om indien nodig te overleggen met mijn revalidatiearts over mijn medische gegevens voor deelname aan de studie.
- Ik wil meedoen aan dit onderzoek.

Naam proefpersoon:

Handtekening: Datum : __ / __ / __

-----------------------------------------------------------------------------------------------------------------

Ik verklaar dat ik deze proefpersoon volledig heb geïnformeerd over het genoemde onderzoek.

Als er tijdens het onderzoek informatie bekend wordt die de toestemming van de proefpersoon zou kunnen beïnvloeden, dan breng ik hem/haar daarvan tijdig op de hoogte.

Naam onderzoeker (of diens vertegenwoordiger):

Handtekening: Datum: __ / __ / __

-----------------------------------------------------------------------------------------------------------------

*De proefpersoon krijgt een volledige informatiebrief mee, samen met een kopie van het getekende toestemmingsformulier.*
